# Ephrin-B3 supports glioblastoma growth by inhibiting apoptosis induced by the dependence receptor EphA4

**DOI:** 10.18632/oncotarget.16077

**Published:** 2017-03-10

**Authors:** Amélie Royet, Laura Broutier, Marie-May Coissieux, Céline Malleval, Nicolas Gadot, Denis Maillet, Lise Gratadou-Hupon, Agnès Bernet, Pascale Nony, Isabelle Treilleux, Jérôme Honnorat, Daniel Liebl, Laurent Pelletier, François Berger, David Meyronet, Marie Castets, Patrick Mehlen

**Affiliations:** ^1^ Apoptosis, Cancer and Development Laboratory-Equipe labellisée ‘La Ligue’, LabEx DEVweCAN, Centre de Cancérologie de Lyon, INSERM U1052-CNRS UMR5286, Université de Lyon, Centre Léon Bérard, Lyon, France; ^2^ Netris Pharma, Lyon, France; ^3^ Lyon Neurosciences Research Center, Neuro-Oncology and Neuro-Inflammation Laboratory, INSERM UMR1028, CNRS UMR5292, Université de Lyon, Lyon, France; ^4^ Research Pathology, Department of Translational Research and Innovation, Centre Léon Bérard, Lyon, France; ^5^ University of Miami Miller School of Medicine, The Miami Project to Cure Paralysis, Miami, Fl, USA; ^6^ Grenoble Institut des Neurosciences, Nanomedicine and Brain Laboratory, INSERM U 836, BP 170, Grenoble, France; ^7^ Centre de Pathologie et de Neuropathologie Est, Hospices Civils de Lyon, Lyon, France

**Keywords:** Ephrin-B3/EphA4, dependence receptors, glioblastoma, angiogenesis, apoptosis

## Abstract

EphA4, an Ephrins tyrosine kinase receptor, behaves as a dependence receptor (DR) by triggering cell apoptosis in the absence of its ligand Ephrin-B3. DRs act as conditional tumor suppressors, engaging cell death based on ligand availability; this mechanism is bypassed by overexpression of DRs ligands in some aggressive cancers. The pair EphA4/Ephrin-B3 favors survival of neuronal progenitors of the brain subventricular zone, an area where glioblastoma multiform (GBM) are thought to originate. Here, we report that Ephrin-B3 is highly expressed in human biopsies and that it inhibits EphA4 pro-apoptotic activity in tumor cells. Angiogenesis is directly correlated with GBM aggressiveness and we demonstrate that Ephrin-B3 also supports the survival of endothelial cells *in vitro* and *in vivo*. Lastly, silencing of Ephrin-B3 decreases tumor vascularization and growth in a xenograft mice model. Interference with EphA4/Ephrin-B3 interaction could then be envisaged as a relevant strategy to slow GBM growth by enhancing EphA4-induced cell death.

## INTRODUCTION

Glioblastoma multiform (GBM) is the most common and malignant brain tumor in adults. Despite development of new therapies and combinations of surgery, radio-and chemo-therapies, GBM remains a therapeutic challenge, the median survival of patients being close to 10 months [1]. Evidences have recently been accumulating in favour of the implication of the subventricular zone (SVZ) neural progenitor cells (NPC) in glioblastoma aetiology [2–8]. SVZ is a brain layer of cells localized around the lateral ventricle, and it is one of the two identified brain stem cell niches [9]. Study of murine models has led to the hypothesis that GBM could originate from NPC of the SVZ that have accumulated oncogenic alterations [2, 4, 8]. It was also shown that recruitment of these progenitor cells may play a role in the aggressive behaviour encountered in GBM [6, 7].

EphA4 is one of the 14 tyrosine kinase receptors linked to the Ephrins family, which notably regulate cell migration and adhesion in a number of biological processes [10–12]. As a tyrosine kinase receptor, EphA4 induces a so-called “forward” downstream signalling when engaged to Ephrins ligands [13]. EphA4 notably functions as a repulsive neuronal guidance cue through binding to Ephrin-B3 [14, 15]. Besides its classical modus operandi, others and we have shown that the pair EphA4/Ephrin-B3 presents some functional particularity. Indeed, EphA4 also functions as a DR [16, 17]: as such and similarly to DCC, UNC5B, TrkA or TrkC [18–20], this receptor is not inactive in the absence of its ligand Ephrin-B3 but rather actively triggers apoptosis [16, 17]. DRs have been shown to act as tumor suppressors thanks to their pro-apoptotic activity; reciprocally, gain of DRs ligands expression can be a selective advantage acquired by cells during tumor escape [21–23]. Along this line, Ephrin-B3 was described as highly expressed in glioblastoma biopsies and notably in invasive tumoral cells [24, 25]. It was also established that apoptotic control driven by the pair EphA4/Ephrin-B3 regulates NPC of the SVZ. Indeed, decrease in NPC number was observed in SVZ from Ephrin-B3 knock-out mice and associated with increase in apoptosis. Reciprocally and consistently with the DR paradigm, reverse phenotype was observed in the same brain area of EphA4 knock-out mice. These observations prompted us to study the apoptotic/survival activity exerted by the pair EphA4/Ephrin-B3 in gliomagenesis.

Here, we first confirmed that Ephrin-B3 is highly expressed in a large fraction of human GBM tumors. We showed that Ephrin-B3 acts as a direct survival factor for GBM tumoral cells *in vitro*, through inhibition of EphA4-DR activity. GBM are highly vascularized tumors, in which tumoral angiogenesis has been directly correlated with spreading and relapse [26]. Since Eph-Ephrins family members have been involved in angiogenesis regulation, we wondered whether the pair EphA4/Ephrin-B3 could stabilize tumoral vessels network. We observed that Ephrin-B3 promotes endothelial cell survival and thus angiogenesis by blocking EphA4-induced endothelial cell death, both *in vitro* and *in vivo* in zebrafish. Lastly, we provide evidence that in GBM, Ephrin-B3 favors tumor growth by inhibiting EphA4-induced endothelial cell death and we then propose to use this trait in a therapeutic perspective.

## RESULTS

### Ephrin-B3 is highly expressed in glioblastoma and acts as a survival factor for tumoral cells via inhibition of EphA4-induced cell death

**Figure 1:**
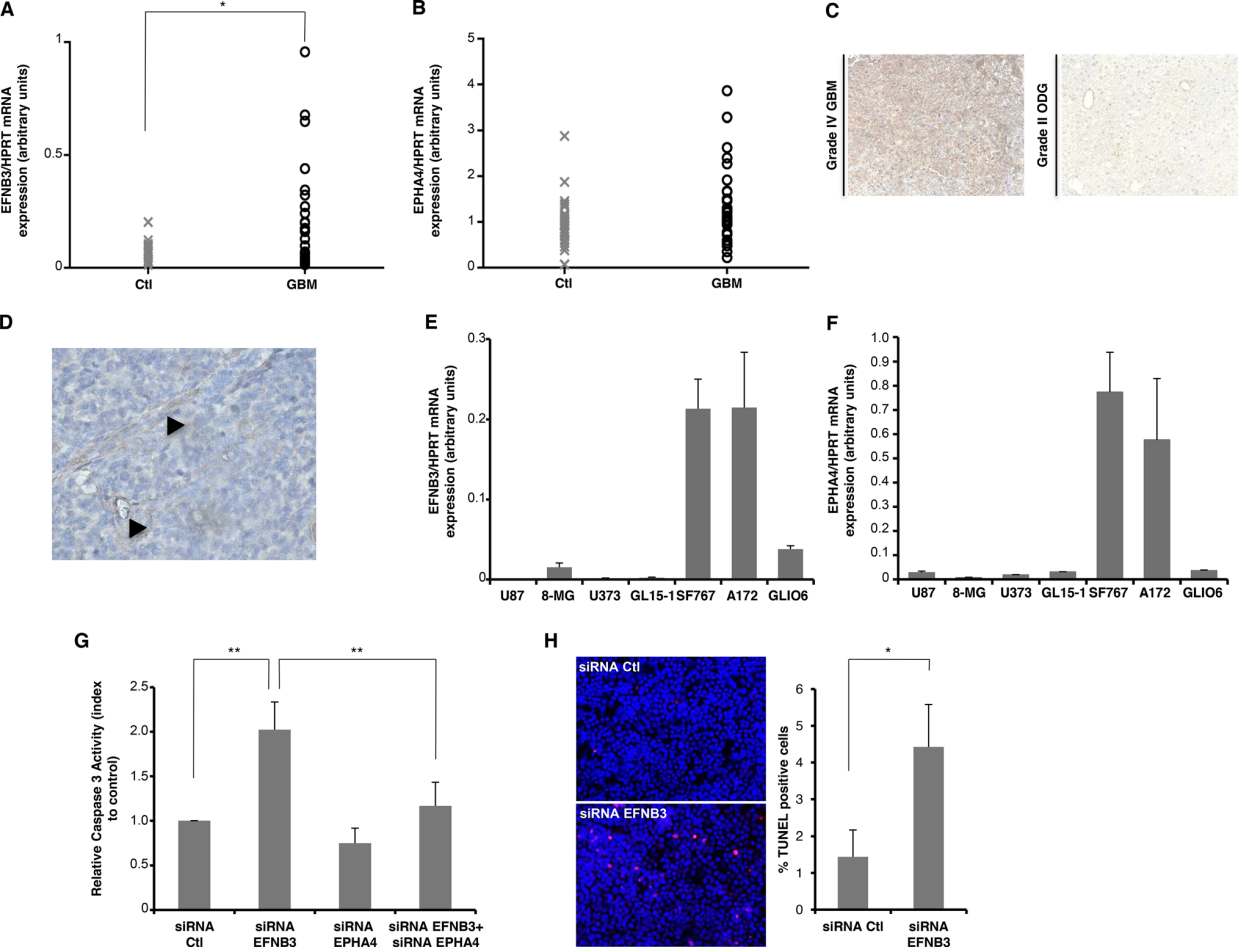
EphrinB3 is highly expressed in glioblastoma tumors and acts as a survival factor for glioblastoma cells, via inhibition of EphA4-induced cell death (**A**, **B**) Total RNA from GBM biopsies collected during curative resectional surgery in the Neurosurgery division of Grenoble hospital (national ethics approval AC-2010-1129) was used to perform Q-RT-PCR quantification, relatively to *HPRT* housekeeping gene expression level. Results are presented for each sample as mean level in three independent experiments. (a) EFNB3 mRNA level is significantly increased in GBM biopsies (*n* = 31) as compared to non-tumoral brain samples (*n* = 28; *p* = 0.01, *t-test*). (b) EphA4 is expressed at similar level in both normal and tumoral samples. No significant correlation with EFNB3 expression was observed. (**C, D**) Analysis of Ephrin-B3 expression in glioma by immunohistochemistry. (c) Representative images of Ephrin-B3 expression in glioma. Five out of seven high-grade glioblastoma (GBM) were positive for Ephrin-B3 expression (left panel) whereas all low-grade glioma, such as oligodendroglioma (ODG), were negative (*n* = 3/3, right panel). (d) Representative images of Ephrin-B3 positive staining in hyperplastic vascular area before tumoral microvessels proliferation (arrowhead) (**E**, **F**) Expression of Ephrin-B3 and EphA4 was measured by Q-RT-PCR on total RNA of 7 cell lines, using *HPRT* housekeeping gene as a standardization control. Results are presented as means +/− std of three independent experiments. (e) EFNB3 is detected in 4 cell lines and is notably high in A172 and SF767 GBM cell lines. (f) EphA4 is also highly expressed in A172 and SF767 GBM cell lines. (**G**, **H**) Silencing of Ephrin-B3 in SF767 GBM tumor cells is sufficient to induce apoptosis and this effect is blocked by co-silencing of EphA4, consistently with DR functioning model. Data are means+/−std of three independent experiments. *p < 0.05; **p < 0.01; *U-test*. (g): Caspase-3 activity measurement and (h): TUNEL assay.

We analyzed Ephrin-B3 expression level in a panel of 31 GBM biopsies by Q-RT-PCR. We confirmed that this gene is highly expressed in tumoral samples, as compared to that observed in 28 non-tumoral white-matter brain biopsies (Figure 1A; *p* = 0.01). Mean expression level of Ephrin-B3 is increased by 2.5 fold, ranging from 0.077 ± 0.047 in control samples to 0.192 ± 0.222 in GBM tumoral biopsies, with 42% of GBM patients presenting a value superior to twice controls mean. No significant change was observed in EphA4 expression profile between normal and tumoral samples (Figure 1B). We analysed Ephrin-B3 expression at the protein level by immunohistochemistry, on 7 glioblastoma WHO grade IV and 3 glioma WHO grade II–II (one oligodendroglioma grade II, one astrocytoma grade II and one astrocytoma grade III). Ephrin-B3 expression level was moderate (*n* = 1) to high (*n* = 4) in more than 70% of glioblastoma samples, whereas its expression is rather low in all low-grade glioma tested (Figure 1C). Interestingly, expression is notably focally high in perivascular area (Figure 1D).

We then aimed to determine whether high expression level of Ephrin-B3 in glioblastoma biopsies could represent a selective advantage for tumoral cells. Expression of Ephrin-B3 was screened in several GBM cell lines: 4 out of 7 cell lines were positive for Ephrin-B3 expression (Figure 1E). The SF767 and A172 cell lines, which show both high Ephrin-B3 and EphA4 expression levels, were selected for further experiments (Figure 1E–1F). Silencing of Ephrin-B3 expression by siRNA leads to an increase in apoptosis of SF767 cells, as shown by caspase 3 activity measurement and TUNEL assay (Figure 1G–1H and Supplementary Figure 1A). Moreover, simultaneous co-silencing of EphA4 is sufficient to prevent induction of apoptosis in Ephrin-B3 silenced SF767 (Figure 1G–1H and Supplementary Figure 1B). Similarly, apoptosis induced by Ephrin-B3 silencing is also reversed by EphA4 co-silencing in A172 cells, whereas it has no impact on U87 Ephrin-B3-negative cells (Supplementary Figure 1A–1D). Thus, in agreement with the DR paradigm, Ephrin-B3 behaves as a survival factor for GBM tumoral cells *in vitro*, by blocking death induced by its unbound EphA4 DR.

### Ephrin-B3 promotes angiogenesis through inhibition of EphA4-induced cell death

**Figure 2:**
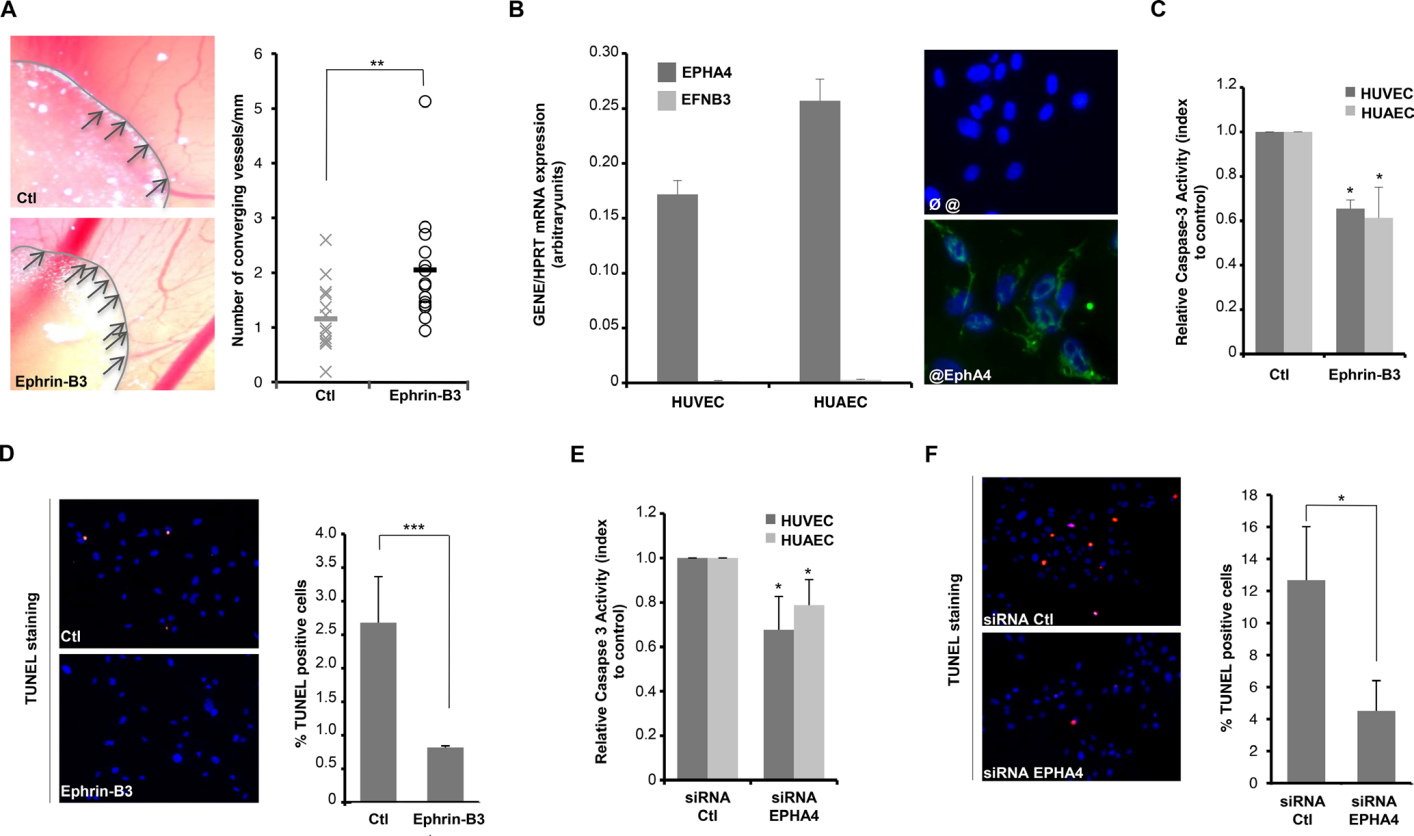
Ephrin-B3 promotes angiogenesis *in vitro* through inhibition of EphA4-induced cell death (**A**) Ephrin-B3 favors angiogenesis in a CAM assay. Angiogenesis is quantified three days after as the ratio of number of vessels converging to the plug to its perimeter (*n* = 14 eggs per condition). Straight lines indicate means. **p < 0.01; *U-test*. (**B**) HUVEC and HUAEC express EphA4 receptor but not Ephrin-B3, as measured by Q-RT-PCR (left panel; results are presented as mean +/− std of 3 independent quantifications) and immunostaining (right panel; upper picture shows control without primary antibody. (**C**) Ephrin-B3 over-expression prevents apoptosis in HUVEC/HUAEC as measured by caspase-3 activity. Data are means +/− std of three independent experiments. *p < 0.05; *U-test*. (**D**) Ephrin-B3 over-expression inhibits apoptosis in HUVECs, as measured by TUNEL staining. TUNEL-positive cells were quantified in blind on 2 independent fields in each condition. Results are presented as mean +/− sem of 3 independent experiments. ***p < 0.001; *U-test*. (**E**) EphA4 silencing by siRNA is associated with a decrease in caspase-3 activity in HUVEC/HUAEC. Relative index of caspase-3 activity is expressed as mean +/− std. *p < 0.05; *U-test*. (**F**) EphA4 silencing prevents apoptosis in HUVECs, as measured by TUNEL staining. TUNEL-positive cells were quantified in blind on at least 3 independent fields in each condition. Results are presented as mean +/− sem of 3 independent experiments. *p < 0.05; *U-test*.

GBM are very invasive tumors characterized by intense and aberrant angiogenic activity, which has been inversely correlated to spreading, relapse and overall patients survival [1, 26, 27]. Eph-Ephrins family members regulate angiogenesis and EphA4 is expressed in endothelial cells [28]. We then wondered whether the pair EphA4/Ephrin-B3 could stabilize tumoral vessels network. As a first hint, we analysed the impact of Ephrin-B3 in a chorioallantoic membrane (CAM) assay [29]. HEK293T cells were transfected or not with an Ephrin-B3 encoding plasmid and grafted at the surface of chicken CAM. As shown in Figure 2A, the number of vessels that develop radially towards Ephrin-B3-positive grafts is significantly increased as compared to controls. We then confirmed by Q-RT-PCR and immunostaining that EphA4 is expressed both in HUVEC and HUAEC (Human Vein/Artery Endothelial Cells), whereas Ephrin-B3 expression is low in these cells (Figure 2B and Supplementary Figure 2A). Forced expression of Ephrin-B3 in HUVEC/HUAEC endothelial cells significantly reduces the extent of spontaneous apoptosis observed in settings of serum starvation conditions (Figure 2C–2D and Supplementary Figure 2B). Moreover, silencing of EphA4 by siRNA is also associated with cell death reduction (Figure 2E–2F and Supplementary Figure 2C). Thus, Ephrin-B3 likely favors endothelial cell survival *in vitro*, at least partially by blocking cell death induced by unbound EphA4.

**Figure 3:**
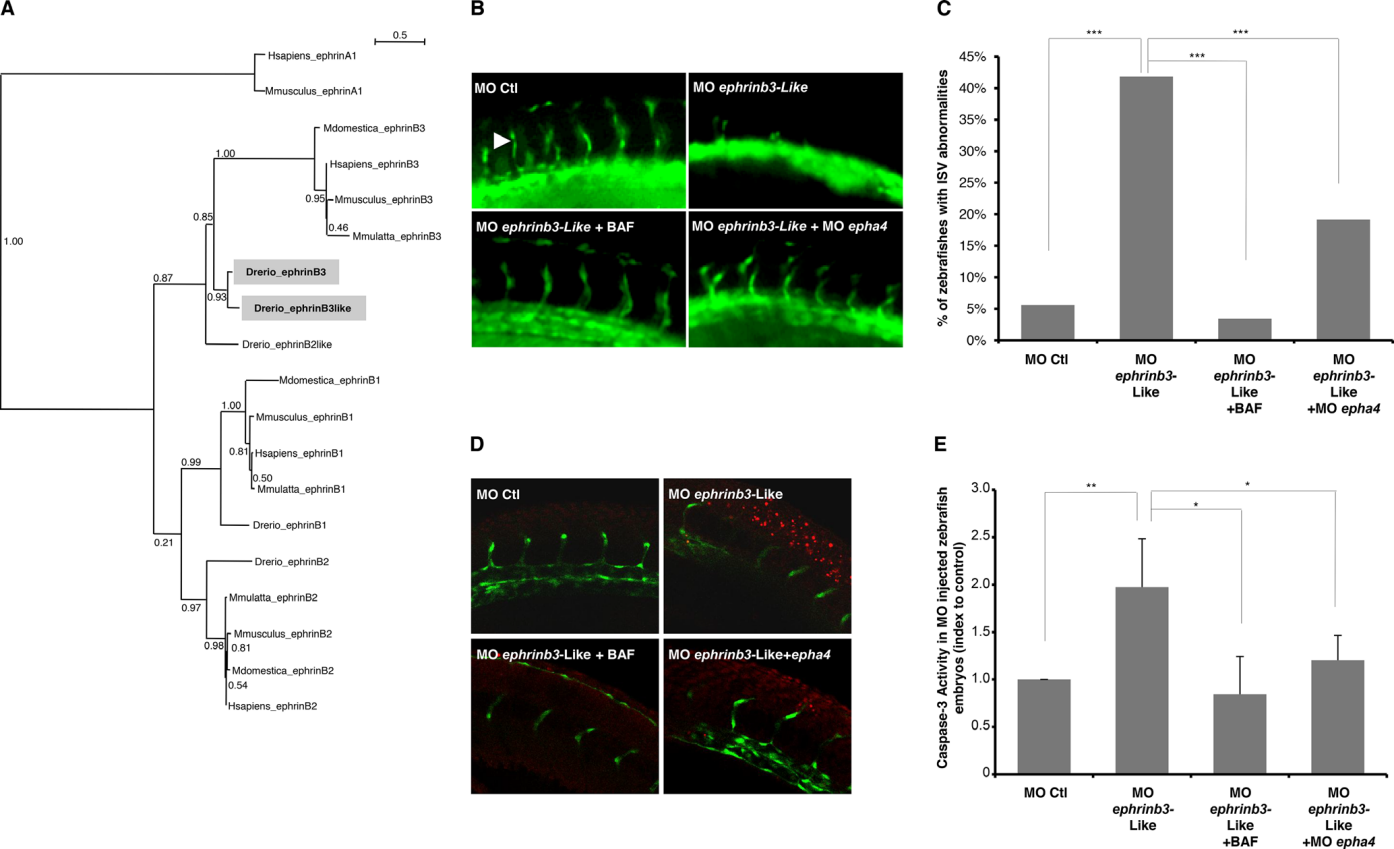
Ephrin-B3 promotes angiogenesis through inhibition of EphA4-induced cell death during zebrafish development (**A**) Phylogenetic tree showing the inferred evolutionary relationships between ephrins members among various biological species. ML on protein sequences, under JTT model, with 100 bootstrap replicates. *EFNB3* orthologs in zebrafish, *ephrinB3-b and ephrinB3-*Like are highlighted. (**B**, **C**) *EphrinB3-Like* silencing triggers defects in intersegmental vessels (ISV) formation, which are significantly rescued by caspases inhibition through BAF treatment or by co-silencing of EphA4. (b) Representative images of trunk vasculature are shown. (c) Quantification of the percentage of embryos with ISV defects in each condition is presented. ***p < 0.001; χ^2^ test. (**D**) BAF treatment or EphA4 silencing are both sufficient to rescue apoptosis in MO *ephrinB3*-Like-injected embryos, as shown by TUNEL staining [36]. Representative images of trunk vasculature are shown. Red dots correspond to apoptotic cells. (**E**) *EphrinB3*-Like silencing also triggers caspase-3 activation in zebrafish embryos, which is significantly rescued by BAF treatment or EphA4 co-silencing. Caspase-3 activity was assessed on whole embryos extracts and is expressed as mean relative index to control. Error bars correspond to standard deviation. *p < 0.05; **p < 0.01; *U-test*.

To further define the role of apoptosis regulation induced by EphA4/Ephrin-B3 pair on angiogenesis *in vivo*, we used the TG(*fli:egfp*)^y1^ transgenic zebrafish model, which targets the expression of GFP specifically in blood vessels and allows direct monitoring of angiogenesis during development [30]. Two *EFNB3* gene orthologs have been described in zebrafish, *ephrinb3-b* and *ephrinb3-Like*, respectively sharing 56% and 51% protein identity with their human ortholog and 69% among each other (Figure 3A). Also overexpression of ephrinb3-b has been suggested to correct vascular defects induced by silencing of the guidance cue *MAX-1* in zebrafish [31], we observed no significant effect on angiogenesis upon silencing of this gene (Supplementary Figure 2D). On the contrary, knock-down of *ephrinb3-Like* impairs intersegmental vessels (ISV) formation, which normally sprout from dorsal aorta along the trunk to form dorsal longitudinal anastomotic vessels (DLAV) (Figure 3B–3C and Supplementary Figure 2D–2E). Indeed, more than 40% of zebrafish embryos knock-down for this *EFBN3* ortholog lack ISV as compared to only 5% of controls (Figure 3B–3C). These vascular abnormalities are associated with an increase in apoptosis in *ephrinb3-Like* knock-down embryos, as shown both by caspase-3 activity measurement and TUNEL assay (Figure 3D–3E). To explore whether these vascular defects could be due to endothelial cells apoptosis induction by EphA4, we treated those injected with *ephrinb3-Like* morpholino with the pan-caspases inhibitor BAF. As shown in Figure 3B–3C, BAF treatment is sufficient to rescue the angiogenic defects detected in *ephrinb3-Like* silenced embryos. Consistently with the DR paradigm, formation of ISV was also rescued by co-silencing of EphA4 (Figure 3B–3C). Altogether, these data support a pro-angiogenic role of Ephrin-B3, which results at least in part from its ability to prevent endothelial cell death induced by unbound EphA4.

### Ephrin-B3 gain of expression in GBM favors tumor growth and vascularization in a mouse xenograft model

**Figure 4:**
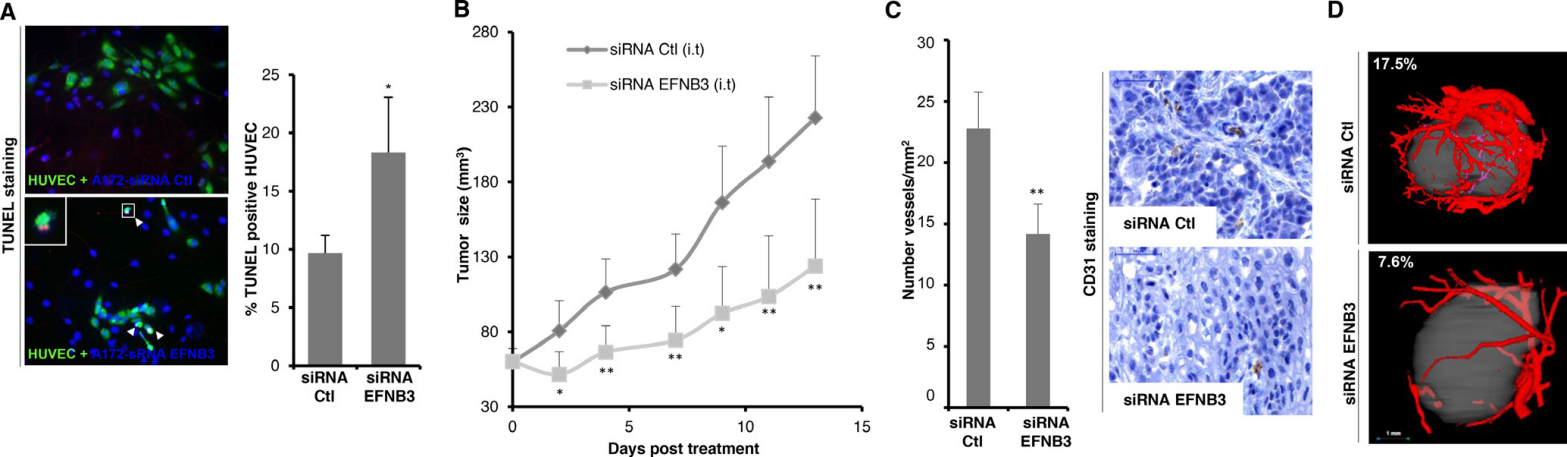
Ephrin-B3 gain of expression in GBM favors tumor's growth and vascularization in a mouse xenograft model (**A**) Silencing of Ephrin-B3 in A172 GBM cells triggers HUVEC cell death in a co-culture assay. Red TUNEL-positive HUVEC cells were quantified in blind on at least 6 fields. Results are presented as mean +/− std of 3 independent experiments. *p < 0.05; *U-test*. (**B**) Silencing of Ephrin-B3 reduces tumoral angiogenesis in a murine xenograft model of GBM. The volume of tumors derived from SF767 was measured during intratumoral (i.t.) injection of control or Ephrin-B3 siRNA diluted in Jet PEI transfectant. Mean tumor volume is indicated (left panel). Datas are mean +/− 90%CIs. *p* values were calculated with a two-side student *t-test*. *p < 0.05; **p < 0.01. (**C**) Microvessels density is significantly decreased in SF767 xenograft tumors silenced for Ephrin-B3 expression. Vessels were stained using an antibody directed against CD31 marker. Representative immunostainings are shown on left panel. Number of vessels on whole slide surface was quantified in blind for each tumor. Results are presented as mean +/− std of 6 different controls or 5 siRNA-EFNB3 treated tumors (right panel). **p < 0.01; *U-test*. (**D**) Vascularization of treated or untreated size-matched tumors was quantified after microfil perfusion of mice using a microcomputed tomography system, and expressed as the percentage of peripheric blood vessels volume to tumor one's. Representative images of one control and one si-EFNB3-treated tumors are shown.

We then aimed to determine if Ephrin-B3 gain of expression observed in GBM could promote tumor growth, both by favoring tumoral cells survival and angiogenesis. We first designed a co-culture assay: GFP-stained HUVEC were co-cultured with A172 GBM cells, silenced or not for Ephrin-B3 expression (Supplementary Figure 3A). As shown on Figure 4A, silencing of Ephrin-B3 in A172 cells leads to a significant increase in HUVEC endothelial cells apoptosis, as measured by TUNEL assay. Thus, *in vitro*, Ephrin-B3 expression by GBM tumor cells favors endothelial cell survival.

We thus moved to an animal model. Since we failed to establish xenografts from A172 cells, SF767 GBM cells, which also express Ephrin-B3 and EphA4 at high levels, were engrafted into *nude* mice. Silencing of EFNB3 through intra-tumoral injection of siRNA coupled to *in vivo* transfectant efficiently decreases EphrinB3 expression in tumoral cells and significantly delays tumor growth (Figure 4B and Supplementary Figure 3B). We then wondered whether this effect could notably result from the impairment of tumoral angiogenesis. We confirmed that EphA4 is indeed expressed in tumor blood vessels (Supplementary Figure 3C). Consistently with the hypothesis of a role of the EphA4/EphrinB3 pair in GBM tumoral angiogenesis, a significant reduction in intra-tumoral microvessels density of mice injected with EFNB3 siRNA was observed (Figure 4C). Quantitative analysis of tumoral angiogenesis by micro-computed imaging was then performed on xenografted tumors after 10 days of treatment (Figure 4D). Mean ratios of peripheral tumor vessels to tumor volumes is decreased in 5 EFNB3 silenced grafts as compared to 6 size-matched control ones, ranging from 11.75 ± 6.00% to 6.22 ± 3.86% respectively.

## DISCUSSION

We show here that Ephrin-B3 behaves as a survival factor for glioblastoma bulk by acting notably on endothelial cells. Even if we cannot discard that Ephrin-B3 could do so via binding to other receptors, we show that its effects are at least partially mediated through inhibition of EphA4-induced cell death, as a rescue of Ephrin-B3 silencing phenotypes is observed upon EphA4 knock-down. Reciprocally, even if other EphA4 ligands, such as Ephrin-B2 and Ephrin-A1, have been respectively shown to exert pro- and anti-oncogenic effects in glioblastoma occurrence and angiogenesis regulation, we failed to observe any significant change in Ephrin-A1, -A2, -A3, -A4, -A5 and -B2 expression upon invalidation of Ephrin-B3 in the tumor xenograft model used here, thereby strengthening the specificity of the effects observed (Supplementary Figure 3D). Ephrins family members are characterized by complex intracellular signalling cascades. Despite the forward signalling induced by Eph receptors upon ligand binding, Ephrin-B ligands are by themselves able to trigger a reverse signalling [32]. This unclassical mechanism of action, in which both ligand and receptor activate downstream cascade of events, coupled with the fact that Eph receptors are not specific of one Ephrin ligand, probably explain the complex role of members of this family in tumorigenesis [33]. We showed here that, in addition, EphA4 cannot be considered as a classical oncogenic growth factors receptor since it may be able to act as a conditional tumor suppressor in absence of Ephrin-B3.

Despite achievements of new aggressive therapies [34], GBM remains a therapeutic challenge. Ephrin-B3 via its ability to block EphA4-induced apoptosis is likely involved in glioblastoma tumorigenesis, since its specific invalidation is sufficient to slow down tumor growth in a xenograft model. Even though this remains to be further studied in an orthotopic model of GBM that would be more relevant than the subcutaneous settings used in this study, these data supports the view of a role of EphA4-induced apoptosis both at the tumoral cell level and at the endothelial cell level. GBM aggressiveness is associated with tumoral angiogenesis, which favours tumor invasion and relapse. Implication of Ephrin-B3 as a survival factor blocking EphA4-induced apoptosis appears crucial in the formation of intersegmental vessels during zebrafish development. Moreover, Ephrin-B3 produced by GBM tumors could enhance endothelial cell survival in their vicinity, thereby favouring tumoral angiogenesis: it may then be speculated that Ephrin-B3 acts as a survival factor in developmental angiogenesis but that this mechanism is also hijacked by some aggressive tumors.

Thus, the work presented here support the development of new anti-cancer therapies based on Ephrin-B3-targeting, in high-Ephrin-B3 expressing cancer cases. It is noteworthy to mention that correlation between Ephrin-B3 expression level and clinical characteristics will have to be defined, to decipher the profile of patients eligible for such a therapy. Because of the complexity of interactions among members of Ephrins/Eph family and of the paradoxical effects that these ligand/receptor pairs may trigger [35], it will be of key importance to develop candidate therapeutics that specifically prevent binding of Ephrin-B3 to EphA4, to avoid off targets effects. Further evaluation will also be required to determine the role of the forward and reverse signalling induced by these Ephrins members in the GBM tumoral context, even if the development of a monoclonal antibody blocking Ephrin-B3/EphA4 interaction may turn as a promising approach.

## MATERIALS AND METHODS

### Patients and tissue samples

Tumor samples were collected from GBM patients during curative resectional surgery in the Neurosurgery division of Grenoble hospital. Samples were frozen (−80°C) next to surgery room and stored for scientific research in a biological resources repository (Centre de Ressources Biologiques, Grenoble Hospital), according to national ethical guidelines. Tissue banking and research conduct were approved by the Ministry of Research (approval AC-2010-1129). Immunohistochemistry was performed on glioma sections from Hospices Civils de Lyon biological resources repository. Anatomopathologic characterization of GBM was performed according to standard international recommendations.

### Plasmid constructs and siRNA

Plasmids encoding Ephrin-B3 was a kind gift from Dr M. Nakada [25]. Human EPHA4 and EFNB3 siRNAs were designed by Dharmacon as a pool of 4 target-specific 20–25 nt siRNAs.

### Endothelial cell culture and transfection

Human Umbilical Vein and Artery Cells (respectively HUVEC and HUAEC) were cultured in complete endothelial cells medium according to manufacturer's instructions (Promocell, passages < 8). HUVEC and HUAEC were transfected using the AMAXA Nucleofector system. 500 000 HUVEC or 1 million HUAEC were suspended in 100 μl HUVEC Nucleofector solution and transfected with 1 μg of plasmid or 20 pmol of siRNA. 24 hours after transfection, cells were transferred into low serum medium and collected for caspase-3 activity assay after 6 hours or for TUNEL assay after 24 hours.

### Glioblastoma cell lines culture

Human glioblastoma cell line A172 and SF767 were respectively cultured in DMEM medium (Gibco^®^, Invitrogen) supplemented with 10% fetal bovine serum without or with 1X MEM Non-Essential Amino-Acids containing Glycine 75 mg/L, L-Alanine 89 mg/L, L-Asparagine 132 mg/L, L-Aspartic acid 133 mg/L, L-Glutamic acid A 47 mg/L, L-Proline 115 mg/L, L-Serine 105 mg/L (Gibco^®^, Invitrogen). A172/SF767 GBM cells were transfected using Interferin (Polyplus Transfection) according to the manufacturer's recommendations. Briefly, cells were plated in six-wells tissue culture plates at a density of 1.10^5^ and transfected 24 hours later with 10 nM of siRNA control (non-targeting 2, Dharmacon), 10 nM of siEFBN3 and 2.5 nM of siEPHA4. 24 hours later, cells were transfected a second time with 10nM of siRNA control (non-targeting 2, Dharmacon) or targeting EFBN3.

### Cell death assays

Cell death assays (TUNEL immunostaining, caspase-3 activity measurement) were performed as described previously [36, 37]. Apoptosis was monitored by measuring caspase-3 activity using Caspase 3/CPP32 Fluorimetric Assay Kit (Gentaur Biovision, Brussel, Belgium). For detection of DNA fragmentation, treated cells were cytospun and Terminal deoxynucleotidyl transferase mediated dUTP-biotin Nick End Labeling (TUNEL) was performed with 300 U/mL TUNEL enzyme (300 U/mL) and 6 μM biotinylated dUTP (Roche Diagnostics, Meylan, France), as previously described [38].

### Quantitative RT-PCR

Total RNA from human biopsies, murine xenografts and cell lines were extracted using the Nucleospin RNAII kit (Macherey- Nagel) and 1 μg was reverse-transcribed using the iScript cDNA Synthesis kit (BioRad). To assay EFNB3, EPHA4 but also EFNA1, EFNA2, EFNA3, EFNA4, EFNA5 and EFNB2 expression, real-time quantitative RT-PCR was performed with specific primers, on a LightCycler 480 apparatus (Roche) using the LightCycler^®^ TaqMan^®^ Master kit (Roche). Reaction conditions for optimal amplifications of each gene, as well as primers selection were determined as already described [39]. The ubiquitously expressed *HPRT* was used as internal controls for human samples. Primers sequences as well as conditions of amplifications are available upon request.

### CAM assay

The chick chorioallantoic membrane (CAM) assay was performed as already described [36]. In brief, HEK293T cells were transiently transfected with Ephrin-B3 or empty vector (pSELECT GFP). 2*10^6^ cells were suspended volume to volume in serum free Matrigel (BDBiosciences) and allowed to solidify to form cell plugs. Plugs were then placed on CAM of 8-days-old chick embryos. Three days after, CAMs were photographed and the angiogenic response was evaluated by measuring the number of vessels that converge to the plug relatively to its perimeter by using AxioVision Release 4.6 software.

### Morpholino knock-down of zebrafish embryos

To knock-down *EphA4* expression, a translation-blocking morpholino (MO) was used as described previously [40]. Knock-down of *ephrinb3-Like* expression was achieved with a translation-blocking morpholino targeting zebrafish ortholog (GeneTools). Specificity of this newly designed MO was assessed by mRNA rescue (Supplementary Figure 2D). In brief, MO-resistant *ephrinb3-Like* allele modified in its 5′UTR was cloned in PCS2+ plasmid (Genscript) and the corresponding mRNA was then obtained using mMESSAGE mMACHINE^®^ (Ambion) *in vitro* transcription kit according to manufacturer's recommendations; 600 pg of *ephrinb3-Like* mRNA was then co-injected with *EFNB3* MO and rescue of *EFNB3* MO-induced phenotype was assessed.

6 ng of MO were injected alone or in combination in the high yolk of one- to four-cells-stage transgenic fli:egfp, or in wild-type zebrafish embryos. Rescue of the phenotype induced by *ephrinb3-Like* silencing was tested by adding 40 μM BAF in the E3 medium 4 hours after morpholinos injection. Caspase-3 activity assay and TUNEL labeling of embryos were performed as described previously [36]. Sequences of morpholinos are available upon request.

### Co-culture assay

HUVECs were plated in 6-well plates at a density of 1.10^5^ and stained using 10 μM green CellTracker^TM^ (Life Technologies). Meanwhile, A172 GBM cells were reverse-transfected using Interferin (Polyplus Transfection) according to the manufacturer's recommendations. Briefly, cells were plated in six-wells tissue culture plates at a density of 1.10^5^ and transfected at the same time with 10 nM of siRNA control (non-targeting 2, Dharmacon) or targeting EFBN3. 24 hours after transfection, 1.10^5^ A172 cells per well were seeded with HUVEC and allowed to adhere for 6 hours in complete endothelial cells medium. Cells were then starved in non-supplemented endothelial cell medium and analyzed by TUNEL assay after 24 hours of co-culture.

### Immunohistochemistry

For histological examination, tissue samples were fixed in 10% buffered formalin and embedded in paraffin. 4 μm thick tissue sections of formalin-fixed, paraffin-embedded tissues were prepared according to conventional procedures. Sections were then stained with hematoxylin and examined with a light microscope. For immunohistochemical analysis, antibodies to EphrinB3, EphA4 (rabbit polyclonal antibody, Abcam) and CD31, an endothelial cell marker (Anaspec, California) were used. An indirect immunoperoxidase technique was applied to deparaffinized tissue sections; the technique was performed using an automated slide stainer (Ventana Discovery, Ventana, Tucson, Ariz., USA).

### Tumor engrafted *nude* mice and tumoral angiogenesis quantification

Seven-weeks-old (20–22 g body weight) female athymic nu/nu mice were obtained from Charles River animal facility. The mice were housed in sterilized filter-topped cages and maintained in a pathogen-free animal facility. SF767 cells were implanted by subcutaneous injection of 5.10^6^ cells in 200 μL of PBS into the right flank of the mice. Once tumors were established (V≈80 mm^3^), mice were treated twice a week by intratumoral injection of 5 μg control siRNA, EFNB3 siRNA and/or EPHA4 siRNA, diluted in JetPEI *in vivo* transfectant. Tumor sizes were measured with a caliper. The tumor volume was calculated with the formula v = 0.5*(length*width^2^). At the end of the treatment, tumors were harvested and fixed in formol. After CD31 staining, microvessels density was quantified in blind for each tumor using Histolab software (*n* = 100 to 300 fields per tumor slide). Vascularization of treated or untreated size-matched tumors was also quantified after microfil perfusion of mice using a microcomputed tomography system, and expressed as the percentage of peripheric blood vessels volume to tumor one's.

## SUPPLEMENTARY MATERIALS FIGURES



## References

[1] Polivka J, Polivka J, Rohan V, Topolcan O, Ferda J (2012). New molecularly targeted therapies for glioblastoma multiforme. Anticancer Res.

[2] S Alcantara Llaguno, Chen J, Kwon CH, Jackson EL, Li Y, Burns DK, Alvarez-Buylla A, Parada LF (2009). Malignant astrocytomas originate from neural stem/progenitor cells in a somatic tumor suppressor mouse model. Cancer Cell.

[3] Galli R, Binda E, Orfanelli U, Cipelletti B, Gritti A, De Vitis S, Fiocco R, Foroni C, Dimeco F, Vescovi A (2004). Isolation and characterization of tumorigenic, stem-like neural precursors from human glioblastoma. Cancer Res.

[4] Holland EC, Celestino J, Dai C, Schaefer L, Sawaya RE, Fuller GN (2000). Combined activation of Ras and Akt in neural progenitors induces glioblastoma formation in mice. Nat Genet.

[5] Ignatova TN, Kukekov VG, Laywell ED, Suslov ON, Vrionis FD, Steindler DA (2002). Human cortical glial tumors contain neural stem-like cells expressing astroglial and neuronal markers in vitro. Glia.

[6] Jafri NF, Clarke JL, Weinberg V, Barani IJ, Cha S (2013). Relationship of glioblastoma multiforme to the subventricular zone is associated with survival. Neuro Oncol.

[7] Lim DA, Cha S, Mayo MC, Chen MH, Keles E, VandenBerg S, Berger MS (2007). Relationship of glioblastoma multiforme to neural stem cell regions predicts invasive and multifocal tumor phenotype. Neuro Oncol.

[8] Wang Y, Yang J, Zheng H, Tomasek GJ, Zhang P, McKeever PE, Lee EY, Zhu Y (2009). Expression of mutant p53 proteins implicates a lineage relationship between neural stem cells and malignant astrocytic glioma in a murine model. Cancer Cell.

[9] Merkle FT, Alvarez-Buylla A (2006). Neural stem cells in mammalian development. Curr Opin Cell Biol.

[10] Flanagan JG, Vanderhaeghen P (1998). The ephrins and Eph receptors in neural development. Annu Rev Neurosci.

[11] Helbling PM, Saulnier DM, Brandli AW (2000). The receptor tyrosine kinase EphB4 and ephrin-B ligands restrict angiogenic growth of embryonic veins in Xenopus laevis. Development.

[12] Wilkinson DG (2001). Multiple roles of EPH receptors and ephrins in neural development. Nat Rev Neurosci.

[13] Noberini R, Koolpe M, Peddibhotla S, Dahl R, Su Y, Cosford ND, Roth GP, Pasquale EB (2008). Small molecules can selectively inhibit ephrin binding to the EphA4 and EphA2 receptors. J Biol Chem.

[14] Kullander K, Butt SJ, Lebret JM, Lundfald L, Restrepo CE, Rydstrom A, Klein R, Kiehn O (2003). Role of EphA4 and EphrinB3 in local neuronal circuits that control walking. Science.

[15] Paixao S, Balijepalli A, Serradj N, Niu J, Luo W, Martin JH, Klein R (2013). EphrinB3/EphA4-mediated guidance of ascending and descending spinal tracts. Neuron.

[16] Furne C, Ricard J, Cabrera JR, Pays L, Bethea JR, Mehlen P, Liebl DJ (2009). EphrinB3 is an anti-apoptotic ligand that inhibits the dependence receptor functions of EphA4 receptors during adult neurogenesis. Biochim Biophys Acta.

[17] Nelersa CM, Barreras H, Runko E, Ricard J, Shi Y, Glass SJ, Bixby JL, Lemmon VP, Liebl DJ (2012). High-content analysis of proapoptotic EphA4 dependence receptor functions using small-molecule libraries. J Biomol Screen.

[18] Llambi F, Causeret F, Bloch-Gallego E, Mehlen P (2001). Netrin-1 acts as a survival factor via its receptors UNC5H and DCC. EMBO J.

[19] Mehlen P, Rabizadeh S, Snipas SJ, Assa-Munt N, Salvesen GS, Bredesen DE (1998). The DCC gene product induces apoptosis by a mechanism requiring receptor proteolysis. Nature.

[20] Nikoletopoulou V, Lickert H, Frade JM, Rencurel C, Giallonardo P, Zhang L, Bibel M, Barde YA (2010). Neurotrophin receptors TrkA and TrkC cause neuronal death whereas TrkB does not. Nature.

[21] Castets M, Broutier L, Molin Y, Brevet M, Chazot G, Gadot N, Paquet A, Mazelin L, Jarrosson-Wuilleme L, Scoazec JY, Bernet A, Mehlen P (2012). DCC constrains tumour progression via its dependence receptor activity. Nature.

[22] Mazelin L, Bernet A, Bonod-Bidaud C, Pays L, Arnaud S, Gespach C, Bredesen DE, Scoazec JY, Mehlen P (2004). Netrin-1 controls colorectal tumorigenesis by regulating apoptosis. Nature.

[23] Mehlen P, Delloye-Bourgeois C, Chedotal A (2011). Novel roles for Slits and netrins: axon guidance cues as anticancer targets?. Nat Rev Cancer.

[24] Hoelzinger DB, Mariani L, Weis J, Woyke T, Berens TJ, McDonough WS, Sloan A, Coons SW, Berens ME (2005). Gene expression profile of glioblastoma multiforme invasive phenotype points to new therapeutic targets. Neoplasia.

[25] Nakada M, Drake KL, Nakada S, Niska JA, Berens ME (2006). Ephrin-B3 ligand promotes glioma invasion through activation of Rac1. Cancer Res.

[26] Reardon DA, Perry JR, Brandes AA, Jalali R, Wick W (2011). Advances in malignant glioma drug discovery. Expert Opin Drug Discov.

[27] Chi A, Norden AD, Wen PY (2007). Inhibition of angiogenesis and invasion in malignant gliomas. Expert Rev Anticancer Ther.

[28] Yao VJ, Ozawa MG, Trepel M, Arap W, McDonald DM, Pasqualini R (2005). Targeting pancreatic islets with phage display assisted by laser pressure catapult microdissection. Am J Pathol.

[29] Ribatti D (2008). Chick embryo chorioallantoic membrane as a useful tool to study angiogenesis. Int Rev Cell Mol Biol.

[30] Lawson ND, In Weinstein BM (2002). vivo imaging of embryonic vascular development using transgenic zebrafish. Dev Biol.

[31] Zhong H, Wu X, Huang H, Fan Q, Zhu Z, Lin S (2006). Vertebrate MAX-1 is required for vascular patterning in zebrafish. Proc Natl Acad Sci USA.

[32] Noberini R, Lamberto I, Pasquale EB (2012). Targeting Eph receptors with peptides and small molecules: progress and challenges. Semin Cell Dev Biol.

[33] Mosch B, Reissenweber B, Neuber C, Pietzsch J (2010). Eph receptors and ephrin ligands: important players in angiogenesis and tumor angiogenesis. J Oncol.

[34] Ohka F, Natsume A, Wakabayashi T (2012). Current trends in targeted therapies for glioblastoma multiforme. Neurol Res Int.

[35] Pasquale EB (2010). Eph receptors and ephrins in cancer: bidirectional signalling and beyond. Nat Rev Cancer.

[36] Castets M, Coissieux MM, Delloye-Bourgeois C, Bernard L, Delcros JG, Bernet A, Laudet V, Mehlen P (2009). Inhibition of endothelial cell apoptosis by netrin-1 during angiogenesis. Dev Cell.

[37] Delloye-Bourgeois C, Brambilla E, Coissieux MM, Guenebeaud C, Pedeux R, Firlej V, Cabon F, Brambilla C, Mehlen P, Bernet A (2009). Interference with netrin-1 and tumor cell death in non-small cell lung cancer. J Natl Cancer Inst.

[38] Ghoumari AM, Wehrle R, Bernard O, Sotelo C, Dusart I (2000). Implication of Bcl-2 and Caspase-3 in age-related Purkinje cell death in murine organotypic culture: an in vitro model to study apoptosis. Eur J Neurosci.

[39] Fombonne J, Bissey PA, Guix C, Sadoul R, Thibert C, Mehlen P (2012). Patched dependence receptor triggers apoptosis through ubiquitination of caspase-9. Proc Natl Acad Sci USA.

[40] Cooke JE, Kemp HA, Moens CB (2005). EphA4 is required for cell adhesion and rhombomere-boundary formation in the zebrafish. Curr Biol.

