# Supplementary material for: Ephrin-B3 supports glioblastoma growth by inhibiting apoptosis induced by the dependence receptor EphA4
Source: Oncotarget. 2017 Mar 10;8(14):23750–9. doi: 10.18632/oncotarget.16077 (PMC5410341; doi:10.18632/oncotarget.16077)
Supplement: Supplementary file 1 [file oncotarget-08-23750-s001.pdf]

Ephrin-B3 supports glioblastoma growth by inhibiting apoptosis induced by the dependence receptor EphA4

Supplementary Materials

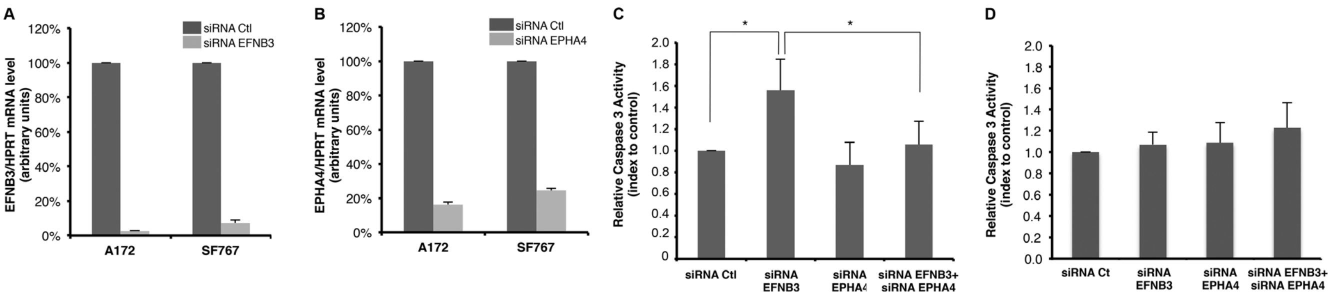

Supplementary Figure 1: Efficiency of genes silencing in GBM cell lines. (**A**, **B**) Specificity of siRNA targeting EFNB3 and EPHA4 was assessed by Q-RT-PCR. Results are presented as mean+/−std of 3 independent quantifications, relatively to *HPRT*. (A) EFNB3 expression is decreased by 97% and 93% respectively in A172 and SF767 cells transfected with specific siRNA targeting EFNB3 as compared to mock transfected cells. (B) EPHA4 expression is decreased by 84% and 76% respectively in A172 and SF767 cells transfected with specific siRNA targeting EPHA4 as compared to mock transfected cells. (**C**) Ephrin-B3 silencing in A172 GBM tumoral cells is sufficient to induce apoptosis and co-silencing of EphA4 blocks this effect, as shown by caspase-3 activity measurement. Data are means+/−std of three independent experiments. *$p < 0.05$; *U*-test. (**D**) Ephrin-B3 silencing does not induce cell death in U87 Ephrin-B3 negative cells, as shown by caspase-3 activity measurement. Data are means+/−std of four independent experiments.

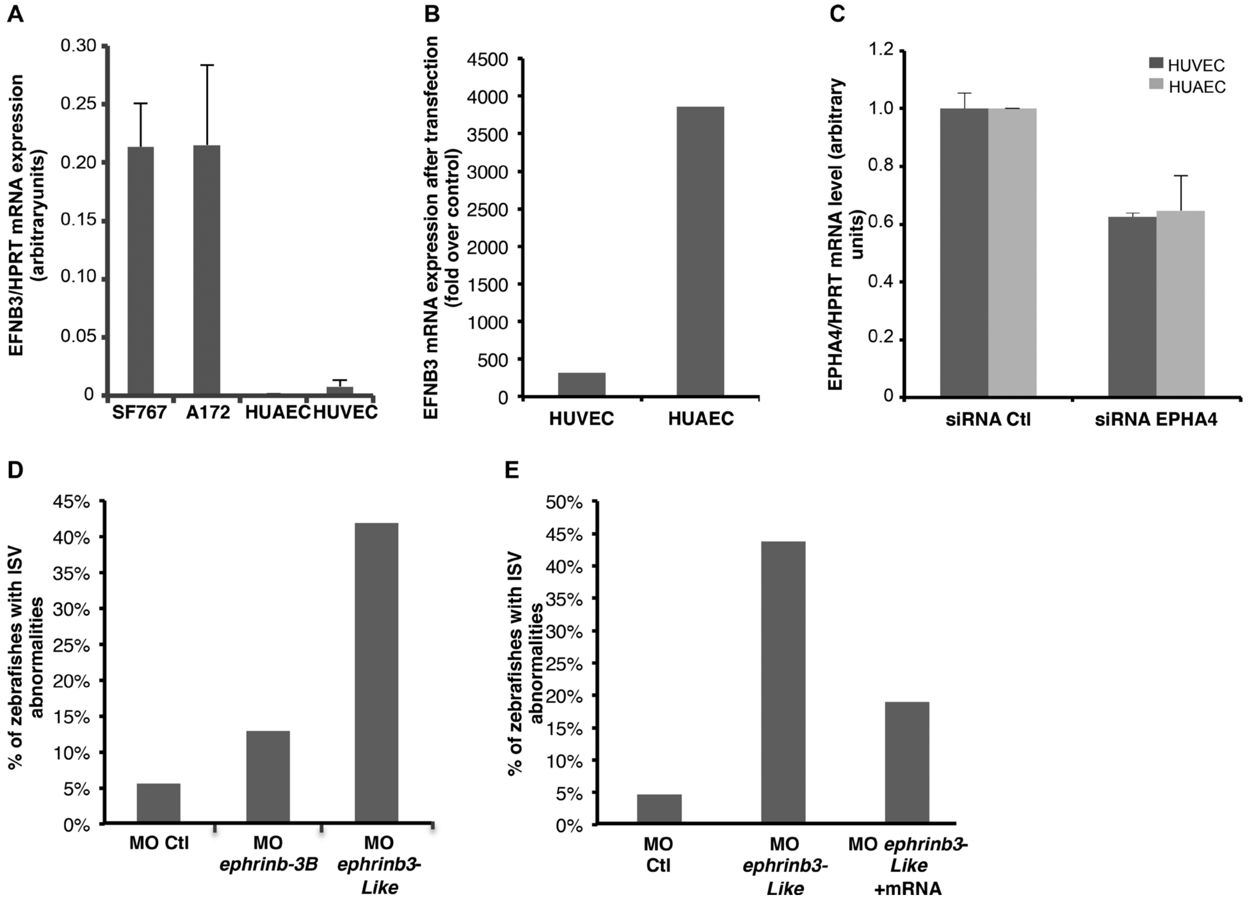

Supplementary Figure 2: Expression and silencing of Ephrin-B3 and EphA4 in endothelial cells. (**A**) Comparison of Ephrin-B3 expression in SF767 and A172 GBM cells with that in HUVEC and HUAEC transfected cells. Results of Q-RT-PCR are mean+/−std of 3 independent quantifications, relatively to *HPRT*. (**B**) Ephrin-B3 expression, quantified relatively to *HPRT* housekeeping gene expression level, is increased in HUVEC and HUAEC transfected with an EFNB3 encoding plasmid as compared to mock transfected cells. (**C**) EPHA4 expression is decreased by 40% in HUVEC and HUAEC transfected with a siRNA targeting EPHA4 as compared to mock transfected cells. Results of Q-RT-PCR are mean+/−std of 3 independent quantifications, relatively to HPRT. (**D**) *Ephrinb3-b* ortholog silencing does not lead to major vascular defects in fli:egfp zebrafish embryos. Embryos were injected at the one- to four-cells stage with morpholinos (MO) targeting *ephrinb3-b* or *ephrinb3*-Like orthologs of mammals EFNB3. Phenotypes were analyzed by fluorescent microscopy 24 hours after injection. (**E**) Co-injection of ephrinb3-Like MO-resistant mRNA restores ISV formation in TG(*fli:egfp*)^{y1} zebrafish embryos. Quantification of the percentage of embryos with ISV defects in each condition is presented.

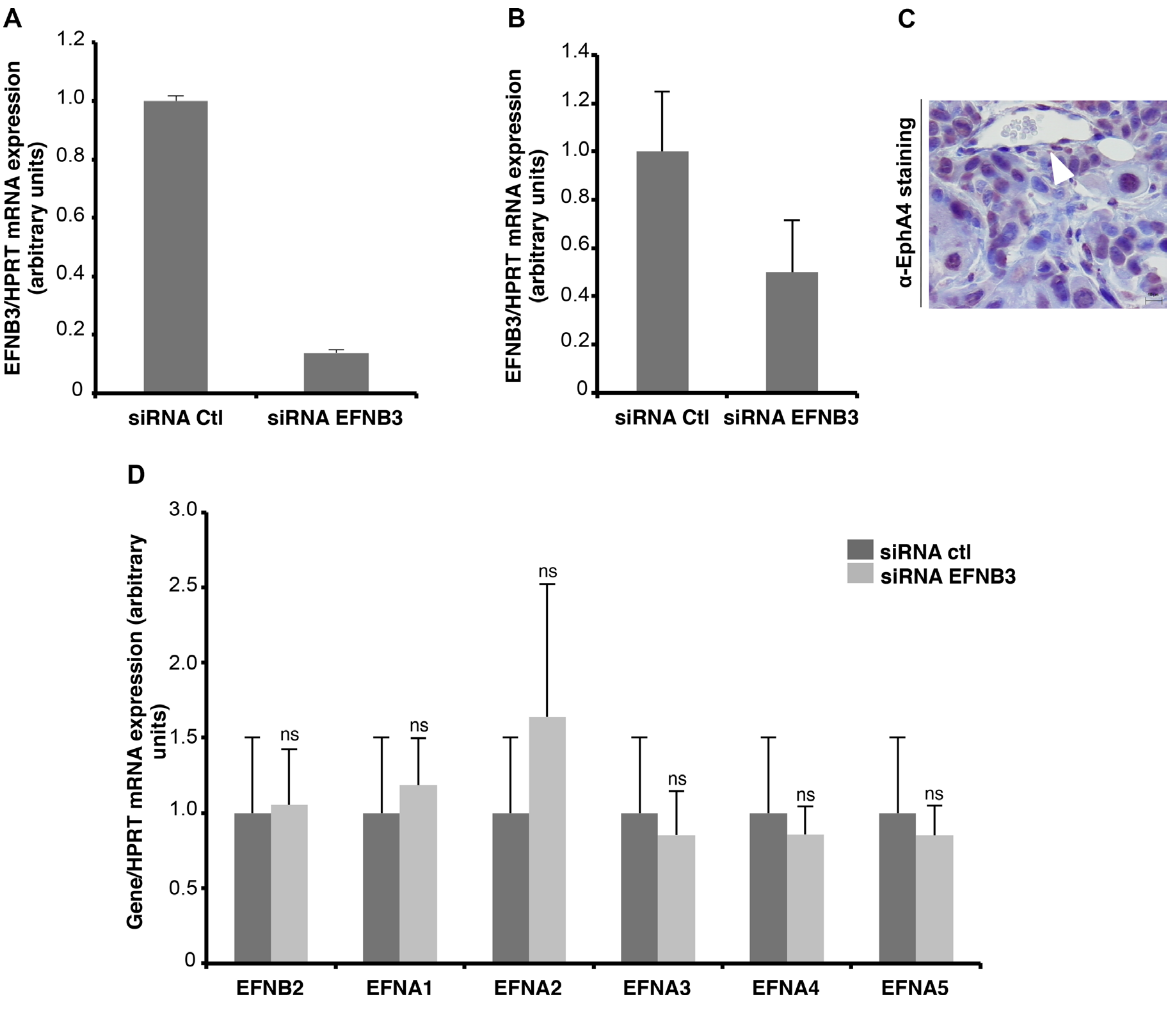

Supplementary Figure 3: Controls and specificity of Ephrin-B3 silencing in GBM cells in co-culture assay and in xenograft experiments. (**A**) Expression of Ephrin-B3 is decreased in A172 GBM cells transfected with a specific siRNA. Results of Q-RT-PCR are presented as mean+/−std of 3 independent quantifications, relatively to *HPRT*. (**B**) Expression of EFNB3 is significantly decreased in SF767 tumors of mice injected with siRNA directed against EFNB3. Ephrin-B3 mRNA level was quantified by Q-RT-PCR to assess the efficiency of in vivo transfection. Results of Q-RT-PCR are mean+/−std of 3 independent quantifications, relatively to *HPRT*. (**C**) Tumoral vessels express EphA4 in a murine xenograft model of GBM. Immunostaining against EphA4 was performed on SF767-derived tumors using a specific antibody. Vessel is indicated by an arrow. (**D**) Expression of EFNA1, EFNA2, EFNA3, EFNA4, EFNA5 and EFNB2 are not significantly changed in SF767 tumors of mice injected with siRNA directed against EFNB3. mRNA levels of ligands were quantified by Q-RT-PCR to check the specificity of *in vivo* transfection. Results of Q-RT-PCR are mean+/−std of 3 independent quantifications, relatively to *HPRT*.
